# Broccoli Consumption and Risk of Cancer: An Updated Systematic Review and Meta-Analysis of Observational Studies

**DOI:** 10.3390/nu16111583

**Published:** 2024-05-23

**Authors:** Eduard Baladia, Manuel Moñino, Eulogio Pleguezuelos, Giuseppe Russolillo, Manuel Vicente Garnacho-Castaño

**Affiliations:** 1 Spanish Academy of Nutrition and Dietetics, 31006 Pamplona, Spain; e.baladia@academianutricion.org (E.B.); mmonyino@academianutricion.org (M.M.); g.russolillo@academianutricion.org (G.R.); 2 Spanish Biomedical Research Center in Physiopathology of Obesity and Nutrition, Carlos III Health Institute, 28029 Madrid, Spain; 3 Department of Physical Medicine and Rehabilitation, Mataró Hospital, Mataró, 08304 Barcelona, Spain; epleguezuelos@csdm.cat; 4 DAFNiS Research Group, Pain, Physical Activity, Nutrition and Health, Campus Docent Sant Joan de Déu, Universitat de Barcelona, Sant Boi de Llobregat, 08830 Barcelona, Spain; 5 Facultad de Ciencias de la Salud, Universidad Internacional de Valencia (VIU), 46002 Valencia, Spain

**Keywords:** cruciferous vegetables, sulforaphane, anticancer agent, cancer prevention, chemopreventive, epidemiological studies

## Abstract

Background: The scientific literature has reported an inverse association between broccoli consumption and the risk of suffering from several types of cancer; however, the results were not entirely consistent across studies. A systematic review and meta-analysis of observational studies were conducted to determine the association between broccoli consumption and cancer risk with the aim of clarifying the beneficial biological effects of broccoli consumption on cancer. Methods: PubMed/MEDLINE, Web of Science, Scopus, Cochrane Library (CENTRAL), and Epistemonikos databases were searched to identify all published papers that evaluate the impact of broccoli consumption on the risk of cancer. Citation chasing of included studies was conducted as a complementary search strategy. The risk of bias in individual studies was assessed using the Newcastle-Ottawa Scale. A random-effects model meta-analysis was employed to quantitatively synthesize results, with the I2 index used to assess heterogeneity. Results: Twenty-three case–control studies (n = 12,929 cases and 18,363 controls; n = 31,292 individuals) and 12 cohort studies (n = 699,482 individuals) were included in the meta-analysis. The results suggest an inverse association between broccoli consumption and the risk of cancer both in case–control studies (OR: 0.64, 95% CI from 0.58 to 0.70, *p* < 0.001; Q = 35.97, *p* = 0.072, I^2^ = 30.49%—moderate heterogeneity; τ^2^ = 0.016) and cohort studies (RR: 0.89, 95% CI from 0.82 to 0.96, *p* = 0.003; Q = 13.51, *p* = 0.333, I^2^ = 11.21%—low heterogeneity; τ^2^ = 0.002). Subgroup analysis suggested a potential benefit of broccoli consumption in site-specific cancers only in case–control studies. Conclusions: In summary, the findings indicate that individuals suffering from some type of cancer consumed less broccoli, suggesting a protective biological effect of broccoli on cancer. More studies, especially cohort studies, are necessary to clarify the possible beneficial effect of broccoli on several types of cancer.

## 1. Introduction

Cancer has become the second leading cause of death worldwide, only surpassed by cardiovascular diseases. However, mortality from cancer is actually higher than from cardiovascular diseases in more-developed countries [1]. In 2020, approximately 10 million patients died from cancer and 19.3 million people were diagnosed with cancer for the first time worldwide, as prevalence continues to rise [2,3].

Since the 1990s, cancer incidence and mortality have tended to decline, while the five-year relative survival rate has increased between 2014 and 2018. Advances in treatment have led to an accelerated decrease in mortality rates in lung, prostate and colorectal cancers in men and in breast and colorectal cancers in women [4].

Researchers have long focused their efforts on identifying multiple risk factors that contribute to the possibility of developing cancer. Tobacco smoking, air pollution, asbestos, alcohol consumption, ultraviolet radiation, Helicobacter pylori infection, lifestyle, excess body weight and poor diet are considered exogenous cancer risk factors associated with a higher incidence of certain types of cancer [5]. In theory, most of these exogenous cancer risk factors are potentially modifiable, which can contribute to preventing and reducing the incidence and mortality of various types of cancer [6].

The role of diet has been the subject of countless epidemiological investigations in cancer prevention. In particular, cruciferous vegetables have been of relevant interest in the scientific literature due to their content associated with anticancer components such as glucosinolates, precursors of isothiocyanates, and indole-3-carbinol [7]. Epidemiological studies and meta-analyses have correlated diets rich in cruciferous vegetables (including broccoli, cauliflower, Brussels sprouts, cabbage, etc.) with a lower risk of several types of cancer, such as lung [8,9], gastrointestinal [10], gastric [7], pancreas [11], colorectal [12,13], bladder [14], renal [15,16], ovarian [17,18], breast [19] and prostate [20] cancers.

Broccoli (*Brassica oleracea* var. *Italica*) is an herbaceous plant of the family *Brassicaceae*, commonly called cruciferous vegetables (*Cruciferae*), characterized by low energy content and high nutritional value due to its fiber, potassium, folate and vitamins C and K contents [21]. Sulforaphane is a compound present in broccoli derived from the hydrolysis of glucoraphanin by the action of myrosinase. This compound is a glucosinolate that is a potent inducer of nuclear factor erythroid 2-related factor 2 (Nrf2), a transcription factor that positively regulates genes associated with the production of antioxidant proteins key to neutralizing oxidative damage. Nrf2 has recently been associated with the modulation of central metabolic pathways [22,23]. Sulforaphane has shown a variety of biological properties that contribute positively to human health. It has been revealed as a chemopreventive and protective agent in various types of cancer, such as colon, gastric, bladder, prostate, breast, skin and lung cancers [24]. Sulforaphane, isolated from broccoli aqueous extract, has shown an inhibitory effect on the damage induced by ultraviolet radiation and the progression of skin cancer [25], as well as decreasing the harmful effects of prostate cancer [26]. In brief, sprouts were boiled in deionized water for 30 min at over 95 °C to extract glucoraphanin. The resulting aqueous extract was cooled to 37 °C, and myrosinase was added, catalyzing the conversion of glucoraphanin to sulforaphane over a 4-h period [26]. The consumption of fresh broccoli is associated with the presence of sulforaphane in plasma and urine [27,28], which is maintained beyond 24 h after consumption. The presence of sulforaphane is greater when it is consumed as a part of vegetables than when taken as an extract. This is possibly due to the presence of myrosinase and other modulating compounds present in fresh broccoli [29,30,31,32], a fact also demonstrated in new varieties of broccoli [33]. The bioavailability of sulforaphane is higher in raw than in cooked broccoli [34]. It also seems that its absorption and bioavailability improve when body mass index is higher than 26 kg/m^2^ [35,36].

These data suggest biological plausibility that would explain and reinforce the possible benefits of broccoli consumption in cancer prevention. Some studies reported positive associations between broccoli consumption and risk in several types of cancer [37,38,39,40,41,42]; however, not all studies were consistent [43,44,45]. To our knowledge, there is no updated systematic review on this topic that includes all types of relevant studies and evaluates broccoli consumption associated with cancer risk. Likewise, not all reviews showed subgroup analyses by study design, which seems relevant for a correct interpretation of the results. Therefore, we conducted a meta-analysis evaluating the association between broccoli consumption and cancer risk with the aim of clarifying the beneficial biological effects of broccoli consumption on cancer.

## 2. Materials and Methods

A systematic review and meta-analysis of observational studies was conducted following the guidelines of Preferred Reporting Items for Systematic reviews and Meta-Analyses (PRISMA 2020) statement [46].

### 2.1. Information Sources and Search Strategies

Systematic searches were performed in electronic databases: MEDLINE via PubMed, Web of Science, Scopus, Cochrane Library (CENTRAL) and Epistemonikos. The initial search strategy was designed for PubMed and adapted to the syntax of the other databases using Polyglot software (available at: https://sr-accelerator.com/#/polyglot; accessed on 14 November 2023) from Systematic Review Accelerator [47].

Search strategies adapted for each database’s syntax are provided in the Supplementary File S1. These strategies were tailored to optimize search efficiency and ensure thorough coverage of relevant literature.

To identify unpublished and ongoing studies, study protocols and scientific conference proceedings and abstracts were also retrieved. Additionally, we carried out forward and backward citation chasing from each included article using the Citation Chaser software (available at: https://estech.shinyapps.io/citationchaser/; accessed on 20 November 2023) [48]. The last search in databases was performed on 22 December 2023.

### 2.2. Eligibility Criteria

The research team used the population, exposure factor, comparator, outcomes, types of study designs (PECOT) approach to specify the eligibility criteria as follows: Population: healthy or sick individuals of any age, sex, ethnicity or country; Exposure: broccoli as a food in any culinary preparation, including raw, cooked and even as beverages; dehydrated broccoli was also included, ensuring that the active components remained intact; studies with broccoli extracts or any active ingredients extracted from broccoli were excluded; Comparison: high consumption of broccoli vs. low or no consumption of broccoli; Outcomes: cancer outcomes were included; and Types of Studies: randomized controlled trials, cohort studies, case–control studies. Systematic reviews, meta-analyses and umbrella reviews were also retrieved as a source of primary studies not retrieved from the database search.

### 2.3. Study Selection Process and Data Extraction

The studies retrieved were managed using ZOTERO software (version 6.0, Corporation for Digital Scholarship, Vienna, VA, USA), and duplicates were manually removed. The resulting library was exported in RIS format and imported to Abstrackr software (available at: http://abstrackr.cebm.brown.edu/account/login; accessed on 12 December 2023) [49].

Blind peer review title and abstract screening was performed by three researchers, EB, MMo and MVG-C, applying the eligibility criteria to identify articles as “included”, “excluded” or “not sure”. Articles tagged as “included” and “not sure” were independently selected for full-text screening by EB, MMo and MVG-C. Discrepancies during the screening were resolved by consensus between EB, MMo and MVG-C. The identification of full-text studies and data extraction were performed at the same time by EB, MMo and MVG-C. The following data were extracted from each study using a piloted form: first author and year, article’s title, objective, study design, sample size, details about the exposure factor, results (association measure and 95% confidence interval) and conclusions.

The search and selection process results were reported using a flow diagram according to the PRISMA 2020 statement [46].

### 2.4. Risk of Bias Assessment

All articles selected for inclusion in this review were case–control and cohort studies. To assess the risk of bias in case–control and cohort studies, EB, MMo and MVG-C made a blinded assessment of each study applying the Newcastle–Ottawa Scale [50]. The scale assesses the study’s methodological quality and covers key aspects of the risk of bias in case–control and cohort studies, and is composed of 4 items that evaluate the selection process, 1 item to assess whether groups are homogeneous (control of confounding factors in the design and/or analysis), and 3 items to assess exposure factors (cases and controls) or outcomes (cohorts) (available at: https://www.ohri.ca/programs/clinical_epidemiology/oxford.asp; accessed on 10 January 2024).

### 2.5. Summary of Outcomes

The characteristics of included studies and main findings were presented in a table of findings, stratifying and organizing the studies based both on cancer outcome and study design, and in line with the methods proposed by the Centre for Reviews and Dissemination [51].

### 2.6. Statistical Analysis

A meta-analysis with Open Metaanalyst software (version 5.26.14; available at: http://www.cebm.brown.edu/openmeta/; accessed on 12 February 2024) was performed using a random effects model (DerSimonian–Laird method), which considers heterogeneity within and between studies, to calculate summary relative risks (RR, cohort studies) and odds ratio (OR, case–control studies) with a confidence interval (CI) of 95% and 3 digits of precision. Forest plots of all possible comparisons were performed. The Cochran’s Q statistic, I^2^ index and tau-squared (τ^2^) were used to evaluate heterogeneity [52]. For the Q statistic, a *p*-value < 0.1 was considered to be representative of statistically significant heterogeneity. For I^2^ index, heterogeneity was classified as follows: 25%—low; 50%—moderate; 75%—high levels of variance. A τ^2^ equal to zero indicates no heterogeneity between studies, and a τ^2^ close to zero indicates lower levels of heterogeneity.

Publication bias was evaluated by Egger’s and Begg’s tests [53,54]. A *p*-value < 0.05 for Egger’s or Begg’s tests was considered representative of significant statistical publication bias.

## 3. Results

### 3.1. Article Selection Process

We identified 3026 articles from databases (772 articles from PubMed/Medline, 893 articles from Web of Science, 1082 articles from Scopus, and 279 from Epistemonikos). After removing duplicates and adding five additional articles identified in published systematic reviews, the complete search strategy resulted in 1369 unique titles and abstracts to be screened.

**Figure 1:**
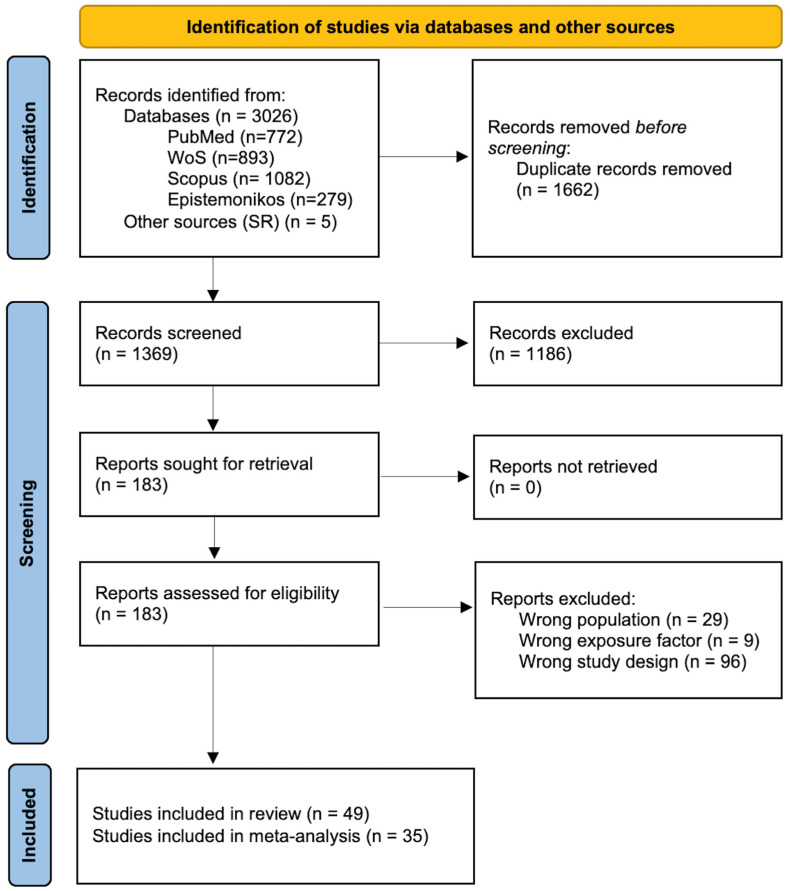
Flowchart of the article selection process.

During the title and abstract screening, researchers reached agreement on including 34 articles, agreed to exclude 1186 articles, and had doubts on 149 papers, mainly due to a lack of data to assess if they met all eligibility criteria. Researchers retrieved 183 full-text to be screened. Finally, 49 articles were selected after complete full-text reading, and 134 were excluded due to not meeting the population, exposure factor or study design criteria. Of the 49 included studies, 14 studies could not be meta-analyzed due to lack of data and data heterogeneity. Finally, 35 studies were meta-analyzed. Figure 1 displays the flow diagram of the search and screening process.

The snowball search using the 49 included studies yielded 172 potential non-screened additional records. From this, 159 articles were excluded after the second-round title and abstract screening, and 13 were selected for complete full-text reading. Finally, all articles were discarded because they did not meet the eligibility criteria or were already included in the review.

### 3.2. Characteristics of the Included Studies

Of the 49 studies included (Table 1), 16 were cohort studies [37,38,43,55,56,57,58,59,60,61,62,63,64,65,66,67] (n = 1,512,760 individuals), and 33 were case–control studies [39,40,41,42,44,45,68,69,70,71,72,73,74,75,76,77,78,79,80,81,82,83,84,85,86,87,88,89,90,91,92,93,94] (n = 18,522 cases and 24,926 controls; n = 43,448 individuals). The association between broccoli consumption and cancer risk was meta-analyzed in 12 cohort studies [37,38,43,55,56,57,59,62,63,64,65,66] (n = 699,482 individuals) and 23 case–control studies [39,40,41,42,44,45,68,69,70,71,72,73,74,75,76,77,78,79,80,81,82,83,84] (n = 12,929 cases and 18,363 controls; n = 31,292 individuals).

High broccoli intake was compared with low consumption. High broccoli intake ranged from daily to weekly consumption, with a minimum frequency of once per week and a maximum of once per day. Low broccoli intake was established from not occurring weekly to three times per month or nonconsumption.

### 3.3. Critical Appraisal

In 21 case–control studies, an independent validation to verify and define the cases was performed (avoiding misclassification bias) [39,68,69,70,71,72,73,77,78,79,80,81,82,83,84,86,87,88,89,90,94]. Fourteen studies had clear representativeness of the cases (selection bias) [39,68,69,71,72,73,77,79,80,81,82,85,86,87], and in 15 studies, controls were selected in hospital environments (Berkson bias) [39,40,42,45,68,70,72,74,75,76,82,84,88,90,93]. As in the cohort studies, the recording of consumption was through self-report surveys or through nonblinded interviews (detection bias) (Table 2).

Only 7 of the 16 cohort studies had adequate representativeness [37,43,58,60,61,64,65] according to the Newcastle–Ottawa Scale; the other nine studies included only health professionals [38,55,56,57,59,62,63,66,67]; therefore, their results could not be inferred to the general population (selection bias). Likewise, in most cohort studies, exposure (consumption of broccoli) was self-reported, with potential detection bias. Uncertainty was also noted regarding whether exposure could vary over time. Long time periods were established for the collection of food consumption data. Most of the other evaluated domains were considered adequate (Table 3).

### 3.4. Outcomes of Case–Control Studies

**Figure 2:**
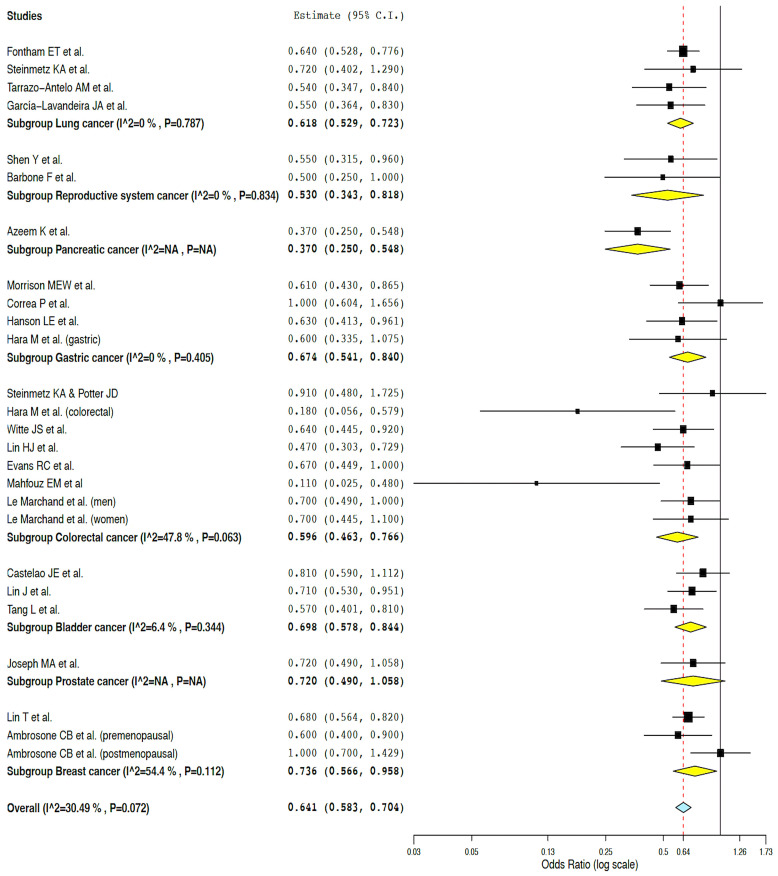
Results of the meta-analysis of case–control studies [39,40,41,42,44,45,68,69,70,71,72,73,74,75,76,77,78,79,80,81,82,83,84].

Of the 33 included case–control studies that evaluated the association between broccoli intake and cancer, 23 could be meta-analyzed [39,40,41,42,44,45,68,69,70,71,72,73,74,75,76,77,78,79,80,81,82,83,84]. Figure 2 shows detailed data from the meta-analysis of case–control studies that evaluated the association between broccoli consumption and various types of cancer.

Overall, the analysis suggested that individuals with higher consumption of broccoli were less likely to suffer from some type of cancer (OR: 0.64, 95% CI from 0.58 to 0.70, *p* < 0.001; Q = 35.97, *p* = 0.072, I^2^ = 30.49%—moderate heterogeneity; τ^2^ = 0.016). In the analysis by cancer subgroups, individuals who consumed more broccoli were less likely to suffer from some site-specific cancers (lung, gastric, colorectal and bladder cancers, *p* < 0.001; reproductive system and breast cancers, *p* = 0.004 and *p* = 0.023, respectively). A low heterogeneity was confirmed for lung cancer (n = 4 studies, Q = 1.06, *p* = 0.788, I^2^ = 0%, τ^2^ = 0.000), reproductive system cancer (n = 2 studies, Q = 0.04, *p* = 0.834, I^2^ = 0%, τ^2^ = 0.000), gastric cancer (n = 4 studies, Q = 2.92, *p* = 0.405, I^2^ = 0%, τ^2^ = 0.000), and bladder cancer (n = 3 studies, Q = 2.14, *p* = 0.344, I^2^ = 6.40%, τ^2^ = 0.002); however, the subgroups of breast cancer and colorectal cancer showed moderate heterogeneity (breast cancer, n = 3 studies, Q = 4.39, *p* = 0.111, I^2^ = 54.45%, τ^2^ = 0.030; colorectal cancer, n = 8 studies, Q = 13.41, *p* = 0.063, I^2^ = 47.79, τ^2^ = 0.057) (Figure 2).

### 3.5. Outcomes of Cohort Studies

**Figure 3:**
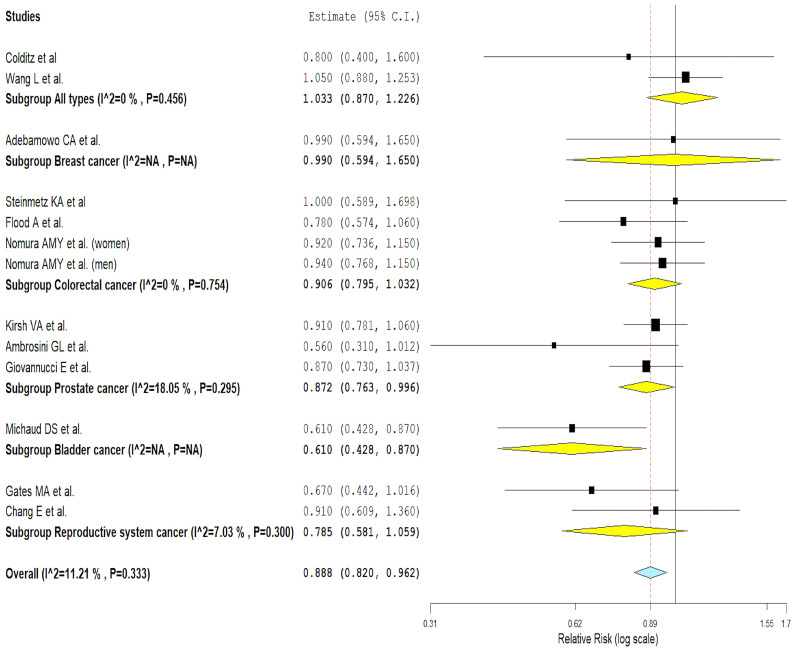
Results of the meta-analysis of cohort studies [37,38,43,55,56,57,59,62,63,64,65,66].

Of the 16 included cohort studies that evaluated the association between broccoli intake and the risk of cancer, only 12 could be meta-analyzed [37,38,43,55,56,57,59,62,63,64,65,66]. Figure 3 presents the detailed data of the meta-analysis of cohort studies that evaluated the association between broccoli consumption and several types of cancer.

The analysis showed that a high consumption of broccoli could be associated with a lower risk of several types of cancer (RR: 0.89, 95% CI from 0.82 to 0.96, *p* = 0.003; Q = 13.51, *p* = 0.333, I^2^ = 11.21%—low heterogeneity; τ^2^ = 0.002). In subgroup analysis, prostate cancer showed a statistically significant inverse association between broccoli intake and cancer risk (*p* = 0.042). However, this association was not statistically significant in colorectal cancer (*p* = 0.136), in reproductive system cancer (*p* = 0.113), and in cancer in general (*p* = 0.713). All cancer subgroups showed low statistical heterogeneity (Figure 3).

**Figure 4:**
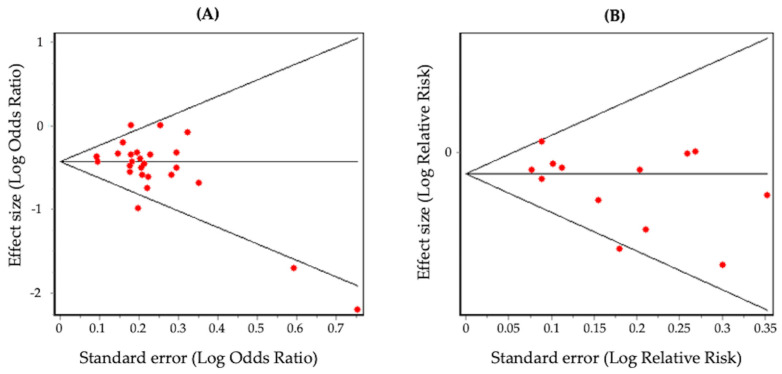
Begg’s funnel plots of case–control (**A**) and cohort (**B**) studies.

In the case–control studies, no evidence of significant publication bias was verified with the Begg’s funnel plot (Figure 4A, *p* = 0.06) or with the Egger’s test (*p* = 0.07). In the cohort studies, no evidence of significant publication bias was observed with the Begg’s funnel plot (Figure 4B, *p* = 0.36) or with the Egger’s test (*p* = 0.08).

## 4. Discussion

To our knowledge, this is the first meta-analysis that evaluates the association between broccoli intake and several types of cancer. According to the results of the meta-analysis, findings from cohort and case–control studies suggested a greater reduction in cancer risk in people who consumed more broccoli compared to those who consumed less or no broccoli; however, there is uncertainty about the robustness of the current available evidence. While more cohort studies are needed to draw more precise conclusions, the results of the case–control studies showed borderline statistical significance with moderate heterogeneity.

Various meta-analyses have verified that the general consumption of cruciferous vegetables is inversely associated with the risk of various types of cancer, such as colorectal cancer, gastric cancer, bladder cancer, pancreatic cancer, breast cancer, and ovarian cancer [7,11,12,14,17,19,20]; however, not all meta-analyses have confirmed this association [95]. Specific analysis for broccoli yielded similar findings, and these results are consistent with the review led by Verhoeven et al., 1996 [96], which considered only case–control studies and suggested that in most of them (56%), the high consumption of broccoli was associated with a lower probability of developing cancer. In our meta-analysis, 17 of 23 case–control studies (73.9%) showed a protective effect of higher broccoli consumption on various types of cancer.

For specific types of cancer, case–control studies suggested that high broccoli consumption was inversely associated with lung or respiratory tract cancer, reproductive cancer, pancreatic cancer, gastric cancer and bladder cancer. Although case–control studies analyzed in colorectal and breast cancer also demonstrated this inverse association, these findings should be interpreted with caution due to the subtle and moderate heterogeneity confirmed in the meta-analyzed studies.

The inverse association observed in case–control studies between greater consumption of broccoli and the risk of suffering from some type of cancer was corroborated in the cohort studies, also observing low heterogeneity in the studies. In this regard, the lower heterogeneity detected in the cohort studies can be attributed, at least in part, to the greater number of case–control studies compared to the cohort studies.

For specific types of cancer, meta-analysis of cohort studies raised doubts about the likely beneficial effect of broccoli consumption in reducing the risk of colorectal cancer and cancer of the reproductive system [59,62,63,64,65]. In alignment with these findings, we did not find an inverse association between broccoli consumption and the risk of suffering from cancer in general (two cohort studies) and breast cancer (one cohort study) (Nurses’ Health Study II; 2005) [43,55,56]. Conversely, a protective benefit of broccoli consumption was observed for bladder cancer [38] and prostate cancer [37,57,66]. These results suggest that broccoli intake could be associated with certain specific cancers. More cohort studies would be necessary to support such claims and further improve the perception of the possible healthy effect of broccoli consumption on specific type of cancer.

Other reviews have previously evaluated the association between broccoli consumption and colorectal cancer with similar findings. In the meta-analysis conducted by Wu et al., 2013, six studies on broccoli were included: three cohort and three case–control studies. The set of studies showed a lower risk of colorectal cancer associated with the consumption of broccoli, although the association was not statistically significant (RR: 0.82; 95% CI 0.65 to 1.02) [13]. By type of study, a non-statistically significant association was observed in the case–control studies (RR: 0.60; 95% CI 0.32 to 1.13) as well as in the cohort studies (RR: 0.91; 95% CI 0.80 to 1.03) [13]. Furthermore, the meta-analysis conducted by Tse et al., 2014 revealed that broccoli intake showed protective benefits against colorectal neoplasia (OR: 0.80, 95% CI: 0.65 to 0.99). It is worth noting that Tse et al. conducted a joint meta-analysis of both case–control and cohort studies, unlike our approach in this meta-analysis [12].

A similar trend was observed in cancer of the reproductive system. The findings from the cohort studies in this review on the impact of broccoli consumption on the risk of reproductive cancer [59,62] are consistent with the results reported by Hu et al., 2015, who observed a 22% reduction in the risk of ovarian cancer with marginal significance for broccoli (RR: 0.78; 95% CI 0.58 to 1.06) [18].

When examining thyroid cancer, a case–control study found no significant association between broccoli consumption and the risk of thyroid cancer [94]. In contrast, findings from a prospective cohort study hinted at a possible positive association between broccoli consumption and thyroid cancer risk in men [60]. However, it is important to note that the results in this study should be interpreted with caution due to the possibility of bias introduced by the presence of other natural goitrogens. Regarding lymphatic cancer, a cohort study suggested that broccoli intake could be associated with a lower risk of non-Hodgkin lymphoma and follicular lymphoma, with a less evident association with diffuse large B-cell lymphoma [61].

As a whole, the results of this review seem to be consistent with the results of previous systematic reviews, both with those that evaluated the impact of the consumption of cruciferous vegetables on cancer [7,11,12,14,17,19,20] and those that assessed the consumption of broccoli [12,13,96]. In this regard, (a) the association between high broccoli consumption and the risk of suffering from various types of cancer showed an inverse trend, that is, the higher the consumption, the lower the risk; (b) the inverse association between higher broccoli intake and the risk of several specific cancers was identified in both case–control and cohort studies; (c) these findings should be interpretated with caution. Case–control studies showed marginal statistical significance with moderate heterogeneity, and further cohort studies are needed.

Several biological mechanisms have been proposed to determine the positive effect of broccoli uptake associated with cancer. The possible protective effect of broccoli could be explained, at least in part, by the chemopreventive and anticancer properties of the metabolites present in this cruciferous plant. Broccoli serves as a significant source of isothiocyanates, small biologically active molecules derived from glucosinolates. Sulforaphane, as an essential compound in broccoli, is an isothiocyanate with notable anticancer and chemopreventive properties [97]. Sulforaphane plays a crucial role in diverse biological processes associated with cancer, including enzymatic detoxification of carcinogens, attenuation of oxidative stress, initiation of cell cycle arrest, promotion of apoptosis, and regulation of the epithelial-to-mesenchymal transition [97,98,99,100,101]. Numerous studies have highlighted sulforaphane’s effectiveness in targeting cancer stem cells across various cancer types, thereby enhancing its potential to prevent drug resistance, metastasis, and tumor recurrence. Sulforaphane has shown its effectiveness against various tumors, including lung cancer, prostate cancer, breast cancer, and colon cancer [97,98,99,100,101,102,103,104].

The observed variance in cancer risk associated with broccoli consumption across studies may predominantly stem from divergent characteristics within studied populations, encompassing variances not only across different cancer types but also across demographic, geographic, cultural, and genetic factors. Additionally, methodological disparities in the assessment of broccoli consumption, including variations in measurement tools and criteria for categorizing high versus low intake levels, could contribute to this heterogeneity. Discrepancies in broccoli preparation methods, notably differences in cooking techniques and the preservation of bioactive compounds, may further amplify this variability. Exposure to high temperatures during cooking can cause the degradation of myrosinase, decreasing its functionality and hindering the synthesis of sulforaphane. Therefore, it is preferable to opt for the consumption of raw broccoli to enhance the bioavailability and protective effects of sulforaphane [97]. Moreover, inconsistencies in the adjustment for confounding factors, despite attempts at standardization across studies, introduce another layer of potential variation, given the divergent types and quantities of confounding factors considered. It is imperative not to discount other potential contributors to disparate findings, including dissimilarities in study methodologies or identified limitations encountered during critical analysis. Finally, it is important to mention that in the studies in which the impact of broccoli consumption was analyzed based on the presence of the GSTM1-null gene polymorphism that is associated with glutathione S-transferase inactivity [76,92], individuals with this polymorphism seemed to benefit more than other subgroups, a finding that could also explain the inconsistency and imprecision of the results.

The primary strength of this study lies in its substantial sample size, comprising 699,482 subjects in the cohort studies and 31,292 participants in the case–control studies. Such a large sample size bestows considerable statistical power, enabling the identification of a robust association between broccoli consumption and the risk of developing various types of cancer.

Several limitations must be considered. It should be considered that the definition of the exposure level varied depending on each study (maximum intake vs. minimum intake of broccoli). Furthermore, the methods for evaluating the level of broccoli intake were heterogeneous due to the types of surveys or tools to measure consumption in each study. The followed-up groups of people were also heterogeneous between the different studies, which could contribute to the inconsistency of the results. As the data from the conducted studies relied on observational methods, it is plausible that the observed inverse association between broccoli consumption and the risk of various cancer types could have been influenced by unmeasured variables or residual confounding factors. In addition, several biases were detected, mainly relating to the representativeness of the cases, selection of controls in hospital environments (Berkson bias), self-reported exposure, nonblinded interviews, and times for the collection of food consumption data.

## 5. Conclusions

This review and meta-analysis may be the most comprehensive to date due to the broad coverage of outcomes for various types of cancer related to broccoli consumption.

From a biological perspective, the consumption of broccoli, regardless of its varieties, shows a protective and chemoprotective effect on cancer and cancer biomarkers. From a methodological perspective, this beneficial effect of broccoli consumption on cancer should be interpreted with caution. Cohort studies should be increased in various specific cancer types, and case–control studies showed subtle moderate heterogeneity.

As a final remark, while broccoli is generally considered a healthy food choice and is associated with various health benefits, including potential cancer-preventive effects, it is important to note that there is generally no significant risk associated with high broccoli consumption for most individuals. However, certain groups may need to exercise caution, including individuals on warfarin medications and people with thyroid issues, allergies/hypersensitivities, or digestive sensitivities [105].

More in-depth studies are warranted to report more-detailed results and stratified results by different cancer types.

## Figures and Tables

**Table 1:** Features and summary of the findings of the studies included in the review evaluating the impact of broccoli consumption on various types of cancer.

Author; Year	Design	Sample (n)	Population/Country/Age	Exposure	Comparison	Outcomes	Effect Size	Follow-Up	Adjustments
Cancer mortality or general cancer incidence									
Colditz et al., 1985 [43]	Cohort study	1271	Men and women/USA 66 years	Broccoli	High intake vs. low intake	Cancer mortality (any type); n = 42 cases	RR: 0.8; 95% CI from 0.4 to 1.6	5 years	Age
Wang L et al., 2009 [55]	Cohort study	38,408	Women/USA ≥ 45 years	Broccoli	High intake vs. low intake	Cancer incidence (any type); n = 3234 cases	RR: 1.05; 95% CI from 0.88 to 1.25	11.5 years	Multivariate
Breast cancer									
Adebamowo CA et al., 2005 [56]	Cohort study	90,630	Women/USA 25–46 years	Broccoli	High intake vs. low intake	Breast cancer	RR (adjusted by age): 1.11; 95% CI: 0.67 to 1.85; RR (multivariable adjustment): 0.99; 95% CI from 0.59 to 1.65	5 years	Multivariate
Lin T et al., 2017 [68]	Cases and controls	1491 cases and 1482 controls	Women/USA 21–97 years	Broccoli	High intake vs. low intake	Breast cancer	OR: 0.68; 95% CI from 0.56 to 0.82 Raw → OR: 0.78; 95% CI from 0.66 to 0.91 Cooked → OR: 0.83; 95% CI from 0.70 to 0.99	1982–1998	Multivariate
Ambrosone CB et al., 2004 [69]	Cases and controls	740 cases and 810 controls	Caucasian women/USA < 50 years, >50 years	Broccoli	High intake vs. low intake	Breast cancer	Premenopausal → OR: 0.6; 95% CI from 0.4 to 1.0 Postmenopausal → OR: 1.0; 95% CI from 0.7 to 1.4	1986–1991	Multivariate
Lung and respiratory tract cancer									
Fontham ET et al., 1988 [39]	Cases and controls	1253 cases; 1274 controls	Men and women/USA	Broccoli	High intake vs. low intake	Lung cancer	OR: 0.64; 95% CI from 0.54 to 0.78	1979–1982	Multivariate
Steinmetz KA et al., 1993 [44]	Cases and controls	138 cases and 2814 controls (random); base cohort (n = 41.837 women)	Women/USA 55–69 years	Broccoli	High intake vs. low intake	Lung cancer; n = 179 cases	OR: 0.72; 95% CI from 0.40 to 1.29	4 years	Multivariate
Tarrazo-Antelo AM et al., 2014 [70]	Cases and controls	371 cases and 496 controls	Men and women/Spain Median > 63 years	Broccoli	High intake vs. low intake	Lung cancer	OR: 0.54; 95% CI from 0.35 to 0.84	2004–2008	Multivariate
García-Lavandeira JA et al. (2022) [72]	Cases and controls	438 cases and 781 controls	Men and women. Never smokers patients/Spain > 66 years	Broccoli	High intake vs. low intake	Lung cancer; adenocarcinoma, n = 289.	OR: 0.55 (0.35–0.83)	2002–2019	Multivariate
Mettlin C. et al., 1989 [85]	Cases and controls	569 cases (355 men/214 women) and 569 controls	Men and women/USA	Broccoli	High intake vs. low intake	Lung cancer	RR: 0.31; 95% CI 0.16 to 0.57	1989	Multivariate/multiple regression
Goodman MT et al., 1992 [86]	Cases and controls	675 cases (463 men and 212 women) and 675 controls	Men and women/USA	Broccoli	High intake vs. low intake	Lung cancer	Women → RR: 2.2; *p* < 0.01) Men → RR: 1.0; *p* = 0.37). Survival of small cell lung cancer in men RR: 2.6; *p* = 0.02	1979–1985	Multivariate
Graham S et al., 1981 [88]	Cases and controls	374 with laryngeal cancer and 381 controls	Men and women/USA	Broccoli	High intake vs. low intake	Lung cancer	NS	1981	-
Digestive tract cancer									
Gastric									
Morrison MEW et al., 2020 [40]	Cases and controls	292 cases and 1168 controls	Men and women/USA	Broccoli	High intake vs. low intake	Gastric cancer	OR: 0.61; 95% CI: 0.43 to 0.86	1992–1998	Multivariate
Correa P et al., 1985 [45]	Cases and controls	391 cases and 391 controls	Men and women/USA	Broccoli	High intake vs. low intake	Gastric cancer	OR: 1.0; 95% CI from 0.7 to 1.7	1985	Multivariate
Hansson LE et al., 1993 [73]	Cases and controls	338 cases and 669 controls	Men and women/Sweden	Broccoli	High intake vs. low intake	Gastric cancer	OR: 0.63; 95% CI from 0.41 to 0.96	Adolescence and 20 years prior to the study	Multivariate
Hara M et al., 2003 [74]	Cases and controls	149 cases and 287 controls	Men and women/Japan 20–70 years	Broccoli	High intake vs. low intake	Gastric cancer	OR: 0.60; 95% CI: 0.34 to 1.08	1998–2002	Multivariate
Graham S et al., 1972 [89]	Cases and controls	228 cases and 228 controls	Men and women/USA	Broccoli	High intake vs. low intake	Gastric cancer	Inverse association, NS	2004–2008	
Colorectal									
Steinmetz KA et al., 1994 [63]	Cohort study	41,837	Women/USA 55–69 years	Broccoli	High intake vs. low intake	Colon cancer; n = 212 cases	RR: 1.0; 95% CI from 0.7 to 1.7	5 years	-
Flood A et al., 2002 [64]	Cohort study	45,490	Women/USA Mean > 60 years	Broccoli	High intake vs. low intake	Colon cancer; n = 485 cases	RR: 0.78; 95% CI from 0.58 to 1.06	7 years	Multivariate/multiple regression
Nomura AM et al., 2008 [65]	Cohort study	85,903 men and 105,108 women	Men and women/USA 45–75 years	Broccoli	High intake vs. low intake	Colorectal cancer	Women → RR: 0.92; 95% CI from 0.75 to 1.15, *p* = 652 Men → RR: 0.94; 95% CI from 0.76 to 1.15, *p* = 652	Average follow-up of 7.3 years	Multivariate/multiple regression
Steinmetz and Potter JD et al., 1993 [71]	Cases and controls	220 cases and 438 controls	Men and women/Australia	Broccoli	High intake vs. low intake	Colon cancer	OR: 0.91; 95% CI from 0.48 to 1.72	1979–1980	Multivariate
Hara M et al., 2003 [74]	Cases and controls	115 cases and 230 controls	Men and women/Japan 20–70 years	Broccoli	High intake vs. low intake	Colorectal cancer	OR: 0.18; 95% CI from 0.06 to 0.58	1998–2002	Multivariate
Witte JS et al., 1996 [75]	Cases and controls	488 cases and 488 controls	Men and women/USA 50–74 years	Broccoli	High intake vs. low intake	Adenomatous polyps	OR: 0.64; 95% CI from 0.44 to 0.92	1991–1993	Multivariate
Lin HJ et al., 1998 [76]	Cases and controls	459 cases and 507 controls	Men and women/USA 50–74 years	Broccoli	High intake vs. low intake	Colorectal adenomas	OR: 0.47; 95% CI of 0.30–0.73;	1991–1993	Multivariate
Evans RC et al., 2002 [77]	Cases and controls	512 cases and 512 controls	Men and women/UK	Broccoli	High intake vs. low intake	Colorectal cancer	Left side colon and rectal cancer (OR: 0.61; 95% CI 0.39 to 0.96); colorectal cancer in general (OR: 0.67; 95% CI 0.45 to 1.00); right colon cancer (OR: 1.00; 95% CI 0.39 to 2.57)	6 years	Univariate
Mahfouz EM et al., 2014 [78]	Cases and controls	150 cases and 300 controls	Men and women/Egypt	Broccoli	High intake vs. low intake	Colorectal cancer	OR: 0.11; 95% CI from 0.01 to 0.48: *p* = 0.03	2010–2011	
Le Marchand et al., 1997 [79]	Cases and controls	Men (698 case–control pairs) Women (494 case–control pairs)	Men and women (different ethnic groups)/USA < 84 years	Broccoli	High intake vs. low intake	Colorectal cancer	Men → OR: 0.7; 95% CI from 0.4 to 1.0; *p* = 0.05 Women → OR: 0.7; 95% CI from 0.4 to 1.1; *p* = 0.18	1987–1991	
Graham S et al., 1978 [87]	Cases and controls	256 colon cancer cases and 783 controls; 330 rectal cancer cases and 628 controls	Men/USA	Broccoli	High intake vs. low intake	Colon and rectal cancer	Inverse association between the consumption of broccoli and the risk of colon cancer, but not rectal cancer, NS	1978	-
Miller et al., 1983 [90]	Cases and controls	194 rectal cancer cases and 542 controls (2nd control series, 535)	Men and women 1st control series without pathologies 2nd series of surgical patients/Canada	Broccoli	High intake vs. low intake	Colon and rectal cancer	Colon cancer OR (men): 1.0; *p*-value: 0.48 OR (women): 1.0; *p*-value 0.43 n = 348 cases Rectal cancer OR (men): 1.0; *p*-value: 0.34 OR (women): 1.2; *p*-value: 0.29 n = 194 cases.	1983	-
Freudenheim JL et al., 1990 [91]	Cases and controls	422 cases (277 men and 145 women) and 422 controls	Men and women/USA	Broccoli	High intake vs. low intake	Rectal cancer	Inversely associated with the risk of rectal cancer in men, but not in women, NS	1978–1986.	-
Slattery ML et al., 2000 [92]	Cases and controls	1579 cases and 1898 controls	Men and women/USA 30–79 years	Broccoli	High intake vs. low intake	Colon cancer	GSTM-1 genotype. OR:1.23; 95% CI from 0.86 to 1.76 for the GSTM1-null genotype OR:0.92; 95% CI from 0.63 to 1.33 for the GSTM1-present genotype OR: 0.30; 95% CI from 0.13 to 0.70; only for the GSTM1-null genotype and age less than 55 years	1991–1994	Multivariate in GSTM1-null genotype
Lin HJ et al., 2002 [93]	Cases and controls	459 cases and 507 controls	Men and women 50–74 years	Broccoli	High intake vs. low intake	Colorectal adenomas	OR 0.41; 95% CI: 0.24 to 0.70 for the GSTM1-null and GSTT1-null genotypes	1991–1993	-
Pancreas									
Azeem K et al., 2016 [41]	Cases and controls	310 cases and 220 controls	Men and women/Czech Republic	Broccoli	High intake vs. low intake	Pancreatic cancer	OR: 0.37; 95% CI from 0.25 to 0.53	2006–2009	-
Liver									
Zhao L et al., 2023 [67]	Cohort study	485,403	Men and women/USA 50–71 years	Broccoli	High intake vs. low intake	Liver cancer	HR: 0.66; 95% CI from 0.54 to 0.81; *p* trend < 0.001.	1995–1996	Multivariate
Urinary tract cancer									
Prostate									
Kirsh VA et al., 2007 [37]	Cohort study	29,361	Men/USA Mean > 62 years	Broccoli	High intake vs. low intake	Prostate cancer	All prostate cancer → RR: 0.91; 95% CI from 0.77 to 1.06 Aggressive prostate cancer → RR: 0.76; 95% CI from 0.59 to 0.99 Extraprostatic cancer → RR: 0.55; 95% CI from 0.34 to 0.89	4.2 years	Multivariate
Ambrosini GL et al., 2008 [57]	Cohort study	1985	Men in a prevention program supplemented with beta-carotene and retinol/Australia. Median 62.6 years	Broccoli	High intake vs. low intake	Prostate cancer. n = 97	RR: 0.56; 95% CI from 0.31 to 1.0	1990–2004	-
Giovannucci E et al., 2003 [66]	Cohort study	47,365	Men/USA < 65 years and ≥65 years	Broccoli	High intake vs. low intake	Total prostate cancer (excluding stage T1a tumors); n = 962	RR: 0.87; 95% CI from 0.73 to 1.05	1986–2000	Multivariate
Joseph MA et al., 2004 [80]	Cases and controls	428 cases and 537 controls	Caucasian Men/USA 45–85 years	Broccoli	High intake vs. low intake	Incident prostate cancer	OR: 0.72; 95% CI from 0.49 to 1.06	1986–1991	Multivariate
Bladder									
Michaud DS et al., 1999 [38]	Cohort study	47,909	Men/USA 40–75 years	Broccoli	High intake vs. low intake	Bladder cancer n = 252 cases.	RR: 0.61; 95% CI from 0.42 to 0.87	10 years	Multivariate
Tang L et al., 2010 [58]	Cohort study	239	Men/USA < 60 years, 60–70 years, and >70 years	Broccoli	High intake vs. low intake	Survival of patients with bladder cancer Cancer deaths n = 101 cases	General death (HR: 0.57; 95% CI 0.39 to 0.83); Disease-specific death (HR: 0.43; 95% CI 0.25 to 0.74)	8 years	Multivariate
Castelao JE et al., 2004 [81]	Cases and controls	1592 cases and controls	Men and women (non-Asians)/USA 25–64 years	Broccoli	High intake vs. low intake	Bladder cancer	OR: 0.81; 95% CI from 0.59 to 1.09	1987–1996	Multivariate
Lin J et al., 2009 [82]	Cases and controls	884 cases and 878 controls	Men and women/USA mean age 64 years cases, 65 years controls	Broccoli	High intake vs. low intake	Bladder cancer, patients who had not received previous chemotherapy or radiotherapy	OR: 0.71; 95% CI from 0.53 to 0.96	1999-Currently ongoing	
Tang L et al., 2008 [83]	Cases and controls	275 cases and 825 controls	Men and women (Predominantly Caucasian)/USA 25–86 years cases; 21–92 years controls	Broccoli	High intake vs. low intake	Bladder cancer	Broccoli raw → OR: 0.57; 95% CI from 0.40 to 0.81 Broccoli cooked → OR: 0.88; 95% CI from 0.65 to 1.20	1982–1998	Multivariate
Reproductive system cancer									
Shen Y et al., 2016 [42]	Cases and controls	600 cases and 236 controls	Women/China 30–50 years	Broccoli	High intake vs. low intake	Uterine fibroids	OR: 0.55; 95% CI from 0.32 to 0.96	2010–2014	Multivariate
Gates MA et al., 2007 [59]	Cohort study	66,940	Women/USA Mean 50–51 years	Broccoli	High intake vs. low intake	Ovarian cancer	RR: 0.67; 95% CI from 0.45 to 1.01	1984–2002	Multivariate
Chang E et al., 2007 [62]	Cohort study	97,275	Women/USA Median age at baseline 50 years	Broccoli	High intake vs. low intake	Ovarian cancer	RR: 0.91; 95% CI from 0.61 to 1.36	1995–2003	Multivariate
Barbone F et al., 1993 [84]	Cases and controls	103 cases and 236 controls	Women/USA	Broccoli	High intake vs. low intake	Endometrial cancer	OR: 0.5; 95% CI from 0.3 to 1.0	1985–1988	Multivariate
Thyroid cancer									
Braganza MZ et al., 2015 [60]	Cohort study	292,477	Men and women/USA Mean: 63.4 years	Broccoli	High intake vs. low intake	Thyroid cancer	HR: 2.13; 95% CI from 1.13 to 3.99; *p* trend < 0.01.	1996–2006	Multivariate
Ron E et al., 1987 [94]	Cases and controls	159 cases and 285 controls	Men and women/USA	Broccoli	High intake vs. low intake	Thyroid cancer	OR: 0.8; *p* trend: 0.20	1987	-
Lymphoid cancer									
Thompson CA et al., 2010 [61]	Cohort study	35,159	Women/USA 55–69 years	Broccoli	High intake vs. low intake	Non-Hodgkin lymphoma (NHL), diffuse large B-cell lymphoma (DLBCL) and follicular lymphoma (FL) n = 415 NHL; 184 DLBCL and 90 FL cases	NHL (RR: 0.72; *p*-value: 0.018). mainly for FL and weaker or not apparent for DLBCL.	1986–2005	Multivariate

Abbreviations: AMI = acute myocardial infarction; CC = coronary heart disease; CI = confidence interval; CVD = cardiovascular diseases; DLBCL = diffuse large B-cell lymphoma; FL = follicular lymphoma; GSTM1 = glutathione S-transferase Mu 1 gene; HR = hazard ratio; n = number of cases; NHL = non-Hodgkin lymphoma; NS = quantitative data not supplied; OR = odds ratio; RCT: randomized controlled trial; RR = relative risk or risk ratio.

**Table 2:** Summary of critical appraisal process based on the Newcastle–Ottawa Scale in case–control studies.

Case–Control Studies								
Author; Year	A.1	A.2	A.3	A.4	B.1	C.1	C.2	C.3
Fontham ET et al., 1988 [39]								
Morrison MEW et al., 2019 [40]								
Azeem K et al., 2016 [41]								
Shen Y et al., 2016 [42]								
Steinmetz KA et al., 1993 [44]								
Correa P et al., 1985 [45]								
Lin T et al., 2017 [68]								
Ambrosone CB., 2004 [69]								
Tarrazo-Antelo AM et al., 2014 [70]								
Steinmetz KA et al., 1993 [71]								
Garcıa-Lavandeira JA et al., 2022 [72]								
Hansson LE et al., 1993 [73]								
Hara M et al., 2003 [74]								
Witte JS et al., 1996 [75]								
Lin HJ et al., 1998 [76]								
Evans RC et al., 2002 [77]								
Mahfouz EM et al., 2014 [78]								
Le Marchand L et al., 1997 [79]								
Joseph MA., 2004 [80]								
Castelao JE., 2004 [81]								
Lin J et al., 2009 [82]								
Tang L., 2008 [83]								
Barbone F et al., 1993 [84]								
Mettlin C et al., 1989 [85]								
Goodman MT et al., 1992 [86]								
Graham S et al., 1978 [87]								
Graham S et al., 1981 [88]								
Graham S et al., 1972 [89]								
Miller AB et al., 1983 [90]								
Freudenheim JL et al., 1990 [91]								
Slattery ML et al., 2000 [92]								
Lin HJ et al., 2002 [93]								
Ron E et al., 1987 [94]								

Color legend: Green: response marked in the tool as the most appropriate option, with a low risk of bias; Red: answer marked in the tool as the least appropriate option; Orange: answer marked as doubtful. Dimensions of case–control studies: A.1. Adequate case definition; A.2. Representativeness of the cases; A.3. Selection of controls; A.4. Definition of controls; B1. Comparability of cases and controls; C.1. Record of exposure (consumption of broccoli); C.2. Same method to record exposure in cases and controls; C.3. Nonrespondent rate.

**Table 3:** Summary of critical appraisal process based on the Newcastle–Ottawa Scale in cohort studies.

Cohort Studies								
Author; Year	A.1	A.2	A.3	A.4	B.1	C.1	C.2	C.3
Kirsh VA et al., 2007 [37]								
Michaud DS et al., 1999 [38]								
Colditz GA et al., 1985 [43]								
Wang L et al., 2009 [55]								
Adebamowo CA et al., 2005 [56]								
Ambrosini GL et al., 2008 [57]								
Tang L et al., 2010 [58]								
Gates MA et al., 2007 [59]								
Braganza MZ et al., 2015 [60]								
Thompson CA et al., 2010 [61]								
Chang ET et al., 2007 [62]								
Steinmetz KA et al., 1994 [63]								
Flood A et al., 2002 [64]								
Nomura AM et al., 2008 [65]								
Giovannucci E et al., 2003 [66]								
Zhao L et al., 2023 [67]								

Color legend: Green: response marked in the tool as the most appropriate option, with a low risk of bias; Red: answer marked in the tool as the least appropriate option; Orange: answer marked as doubtful. Cohort study dimensions: A.1. Representativeness of the exposed cohort (broccoli consumption); A.2. Selection of the unexposed cohort; A.3. Record of exposure; A.4. Verification of outcome not present at the beginning of the study; B1. Cohort comparability; C.1. Evaluation of the outcome; C.2. Sufficient exposure time; C.3. Adequate follow-up of the cohort.

## Data Availability

All data of this study are available from the corresponding author on reasonable request.

## Supplementary Materials

The following supporting information can be downloaded at: https://www.mdpi.com/article/10.3390/nu16111583/s1.
