# Supplementary material for: Broccoli Consumption and Risk of Cancer: An Updated Systematic Review and Meta-Analysis of Observational Studies
Source: Nutrients. 2024 May 23;16(11):1583. doi: 10.3390/nu16111583 (PMC11174709; doi:10.3390/nu16111583)
Supplement: Supplementary file 1 [file nutrients-16-01583-s001.zip › nutrients-3004355-supplementary.pdf]

Supplementary File S1

Database search strategies and results

Pubmed/Medline search strategy

String	Search details	Results
#1	(("Brassica"[Mesh] AND oleracea[tiab]) OR "Brassica oleracea"[tiab] OR broccoli[tiab])	5,385
#2	"Neoplasms"[Mesh] OR Neoplasms[tiab] OR Cancers[tiab] OR Neoplasm[tiab] OR Cancer[tiab] OR Tumor[tiab] OR Tumors[tiab]	4,952,961
#3	#1 AND #2	772

Web of Science search strategy

String	Search details	Results
#1	((ALL=Brassica AND (TI=oleracea OR AB=oleracea)) OR (TI="Brassica oleracea" OR AB="Brassica oleracea") OR (TI=broccoli OR AB=broccoli))	12,482
#2	TI=Neoplasms OR AB=Neoplasms OR TI=Cancers OR AB=Cancers OR TI=Neoplasm OR AB=Neoplasm OR TI=Cancer OR AB=Cancer OR TI=Tumor OR AB=Tumors	3,796,267
#3	#1 AND #2	893

Scopus search strategy

String	Search details	Results
#1	((INDEXTERMS(Brassica) AND TITLE-ABS(oleracea)) OR TITLE-ABS("Brassica oleracea") OR TITLE-ABS(broccoli))	13,590
#2	INDEXTERMS(Neoplasms) OR TITLE-ABS(Neoplasms) OR TITLE-ABS(Cancers) OR TITLE-ABS(Neoplasm) OR TITLE-ABS(Cancer) OR TITLE-ABS(Tumor) OR TITLE-ABS(Tumors)	5,056,405
#3	#1 AND #2	1,082

Epistemonikos search strategy

String	Search details	Results
#1	(title:((Brassica OR broccoli OR cruciferous) AND (Neoplasm* OR Cancer* OR tumor*)) OR abstract:((Brassica OR broccoli OR cruciferous) AND (Neoplasm* OR Cancer* OR tumor*)))	279
